# Supplementary material for: Asynchronous magnetic resonance elastography: Shear wave speed reconstruction using noise correlation of incoherent waves
Source: Magn Reson Med. 2022 Oct 27;89(3):990–1001. doi: 10.1002/mrm.29502 (PMC9792433; doi:10.1002/mrm.29502)
Supplement: Supplementary file 1 — DATA S1 MATLAB code used to generate the numerical simulations of Figure 1 [file MRM-89-990-s002.zip › k-Wave/helpfiles/roundOdd.html]

roundOdd :: Functions (k-Wave)


# roundOdd

Round towards the nearest odd number.

## Syntax

```
x = roundOdd(x)
```

## Description

`roundOdd` rounds each element of x towards the nearest odd integer.

## Inputs

|  |  |
| --- | --- |
| `x` | input value/s |

## Outputs

|  |  |
| --- | --- |
| `x` | value/s of x rounded to nearest odd integer |

## See Also

`round`, `roundEven`
